# Rapid growing mass of the mandible due to an oral metastasis of thymoma: Case report of an extremely rare localization and review of published cases

**DOI:** 10.1016/j.heliyon.2025.e41931

**Published:** 2025-01-13

**Authors:** Francesco Scilla, Cosimo Rupe, Gioele Gioco, Luca Raffaelli, Filippo Lococo, Ciro Mazzarella, Guido Rindi, Romeo Patini, Carlo Lajolo

**Affiliations:** a Head and Neck Department, Fondazione Policlinico Universitario A. Gemelli IRCCS, Rome, Università Cattolica del Sacro Cuore, Rome, Italy; b Thoracic Surgery, Fondazione Policlinico Universitario A. Gemelli IRCCS, Rome, Università Cattolica del Sacro Cuore, Rome, Italy; c UOC di Radioterapia Oncologica, Dipartimento Diagnostica per Immagini, Radioterapia Oncologica ed Ematologia, Fondazione Policlinico Universitario “A. Gemelli” IRCCS, largo A. Gemelli, Rome, Italy; d Institute of Pathology, Fondazione Policlinico Universitario “A. Gemelli”, IRCCS, Rome, Università Cattolica del Sacro Cuore, Rome, Italy

**Keywords:** Neoplasm metastasis (D009362), Oral medicine (D019242), Case report, Literature review, Thymoma (D013945)

## Abstract

**Background:**

Cancer metastases in the oral cavity are relatively uncommon, occurring in approximately 1–3% of malignant cases. Thymomas and thymic carcinomas are the two main tumors that can affect the thymus, a lymphatic gland that plays an important role in regulating the immune system. The aims of this paper are (i) to describe an extremely rare case of thymoma metastasis to the oral cavity (ii) report all published cases of thymic tumor metastases to the oral cavity.

**Case report:**

A 62-year-old Italian male patient was referred to the Oral Medicine Department at the Fondazione Policlinico Universitario A. Gemelli – IRCCS, Rome to evaluate a rapidly growing, non-ulcerated swelling in the left buccal mucosa. After clinical examination and radiological examination, an incisional biopsy was performed under local anesthesia. Thus, a final diagnosis of intraoral metastasis of thymoma was achieve.

A review of the literature of oral metastases due to thymic tumor was also conducted through a systematic search in accordance with PRISMA guidelines, via PubMed, Scopus and CENTRAL engines. Out of 328 articles retrieved, only two articles reported oral metastasis of thymic tumors; and two reports of ectopic thymic carcinoma of the parotid gland were found.

**Conclusions:**

This paper provides a rare case of oral metastasis of thymoma. The present paper reports an almost undescribed condition. Future reports will be useful in increasing knowledge regarding this area. The oral screening of patients with thymic tumors could be useful, considering the relationship between these diseases and oral disorders.

## 1 Introduction

Cancer metastases in the oral cavity are relatively uncommon, occurring in approximately 1–3% of malignant cases and may arise either in soft tissues or bone, and then spread locally [1]. The majority of the oral metastases occur in presence of an advanced disease. Notably, almost 25 % of oral metastases are the initial sign of metastatic spread and 23 % are the first sign of a previously undiagnosed malignant tumor in another location [2]. The age at which oral metastases are diagnosed varies greatly and typically falls between 40 and 70 years without a sex predilection for bone metastases, with a 2:1 ratio for those affecting the soft tissues [3].

The most common location for oral metastases is the mandible, with the molar region being the most frequent site (>50 %), followed by the premolar area (38 %) and the mandibular angle (29 %). In soft tissues, the attached gingiva is the most commonly affected site (60 %), followed by the tongue (18 %) [4].

Thymomas and thymic carcinomas are the two main tumors that can affect the thymus, a lymphatic gland that plays an important role in regulating the immune system. Both neoplasms originate from the epithelial cells of the thymus gland and are more common in adults, with the onset being more common after the age of 50 for thymoma and 40 years for thymic carcinoma [5]. The overall incidence is 0.13 per 100.000 person-years [6].

Thymoma and thymic carcinoma have some similarities in terms of histology, but differ in terms of clinical behavior. Thymomas tend to be less aggressive than thymic carcinomas and have a lower potential for malignancy. Thymoma and thymic carcinoma are classified according either to tumor cell morphology (WHO 2015 classification) or invasiveness into the surrounding tissues (Masaoka-Koga 1994 Staging System) [5] (Table 1).

**Table 1:** WHO histologic classification and Masaoka_Koga staging system.

**Type**	**Histologic description**	**Stages**	**Description**
A	Medullary thymoma	Stage I	Completely encapsulated
AB	Mixed thymoma	Stage IIA	Microscopic invasion through the capsule into surrounding fatty tissue
B1	Predominantly cortical thymoma	Stage IIB	Macroscopic invasion into capsule
B2	Cortical thymoma	Stage III	Macroscopic invasion into adjacent organs
B3	Well-differentiated thymic carcinoma or epithelial thymoma or squamoid thymoma	Stage IVA	Pleural or pericardite implants
C	Heterogeneous thymic carcinoma	Stage IVB	Lymphogenous or hematogenous metastasis to distant (extra thoracic) sites

The aims of this paper are (i) to describe an extremely rare case of thymoma metastasis to the oral cavity and (ii) report all the published cases of thymic tumor metastases to the oral cavity.

## 2 Case report

**Fig. 1:**
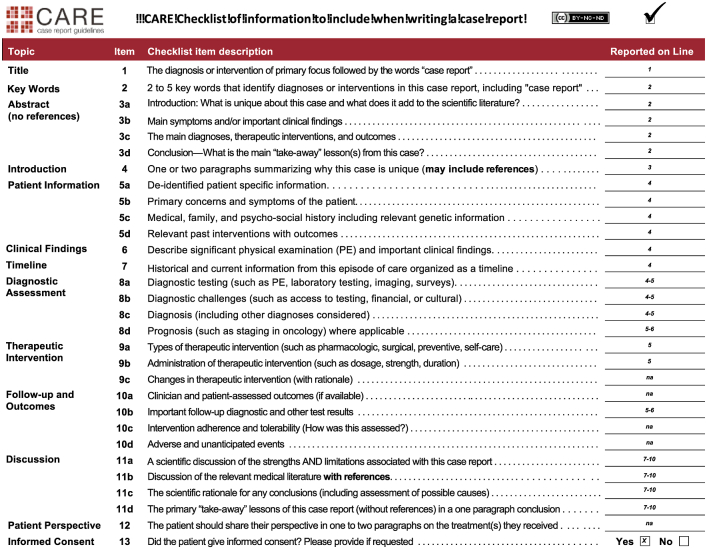
CARE checklist.

The present Case Report has been written in accordance with the CARE Guidelines [7] (Fig. 1).

### 2.1 Patient information

In October 2022, a 62-year-old Italian male patient was referred to the Department of Oral Medicine of the “Policlinico Universitario Agostino Gemelli” Hospital by the Oncology Department, to evaluate a rapidly growing, non-ulcerated swelling in the left buccal mucosa that made it difficult to chew and close his mouth. The patient's medical history, not reporting any smoking or alcohol consumption habit, revealed a thymoma diagnosed in 2019 following endovascular mediastinal biopsy. No other comorbidities were reported. In August 2020, thymectomy was performed, including resection of the right and left anonymous veins, followed by histological diagnosis of a B3 thymoma (WHO 2015) [8] at stage 3 (Masaoka-Koga 1994) [9]. In February 2022, a biopsy was performed in the left supraclavicular area, with a histological examination describing a lymph node metastasis of the thymoma.

### 2.2 Clinical findings

**Fig. 2:**
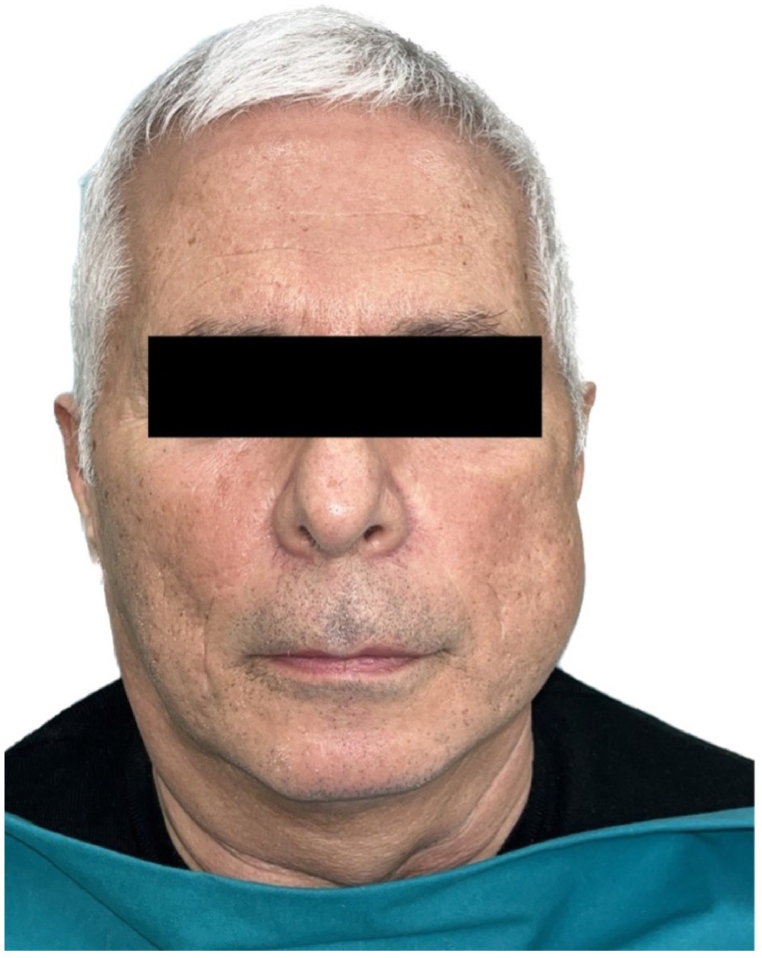
Swelling of the left hemiface.

**Fig. 3:**
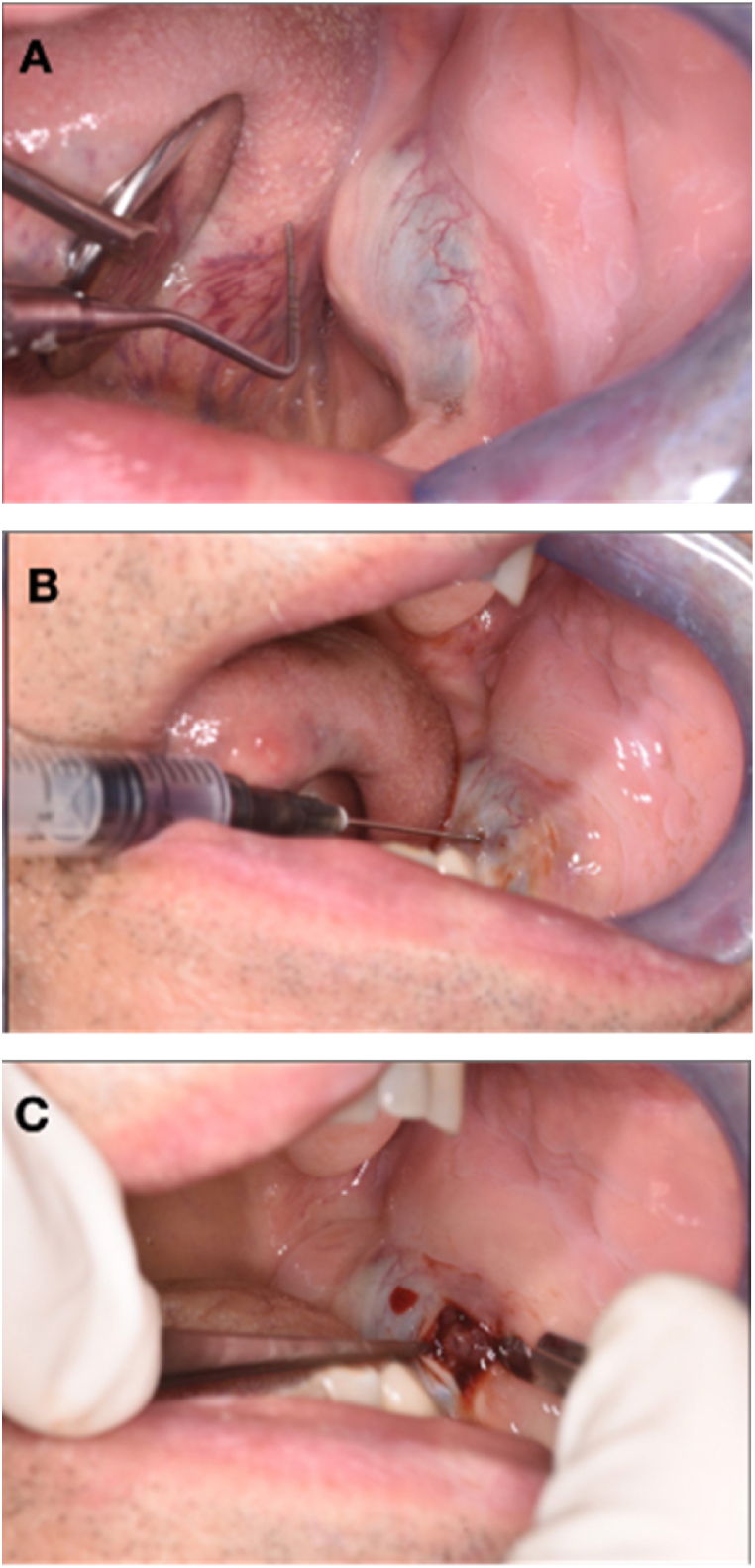
(A) Intraoral view of the lesion; (B) Needle aspiration of the lesion, sent by thin prep technique; (C) Lesion detail.

Extra-oral clinical examination revealed significant swelling of the left hemiface, which was not painful (Fig. 2). Intraoral clinical examination confirmed the presence of swelling in the left mandible, at the level of the mandibular angle in the retromolar trigone area, which was non-ulcerated and completely enclosed within the epithelium, in the absence of pain, neither spontaneous nor provoked, and had a hard-elastic consistency and bluish in color (Fig. 3A). The patient reports lip numbness of the left lower and upper hemilabra, i.e., the part closest to the lesion. The patient reported its recent onset (about 15 days before) and a rapidly worsening trend. The lesion measured approximately 3 cm × 2 cm × 2 cm.

### 2.3 Diagnostic assessment

**Fig. 4:**
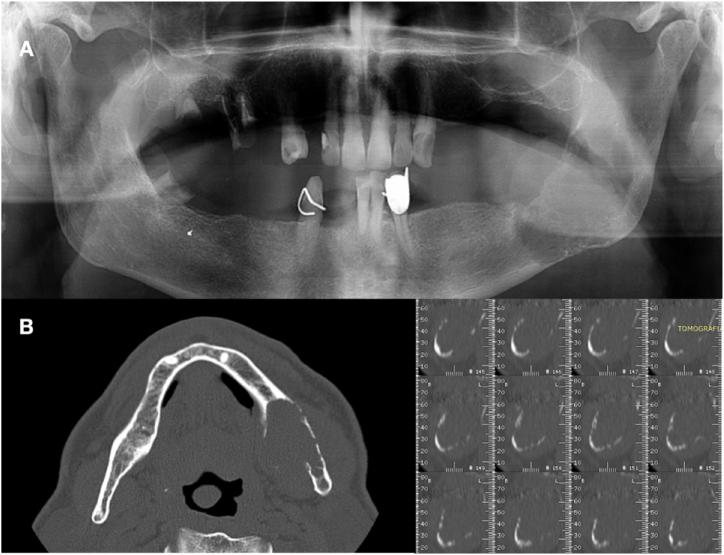
(A) OPT: Large osteolytic area of left mandible is detected; (B) TC dentascan sections.

The first-level radiographic examination (Orthopantomography of the dental arches, Fig. 4A) showed a large area of radiolucency in the left mandibular angle. Therefore, a CT dentascan radiographic examination was requested (Fig. 4B and C), which revealed a large osteolytic area of the left mandible in the posterior region.

**Fig. 5:**
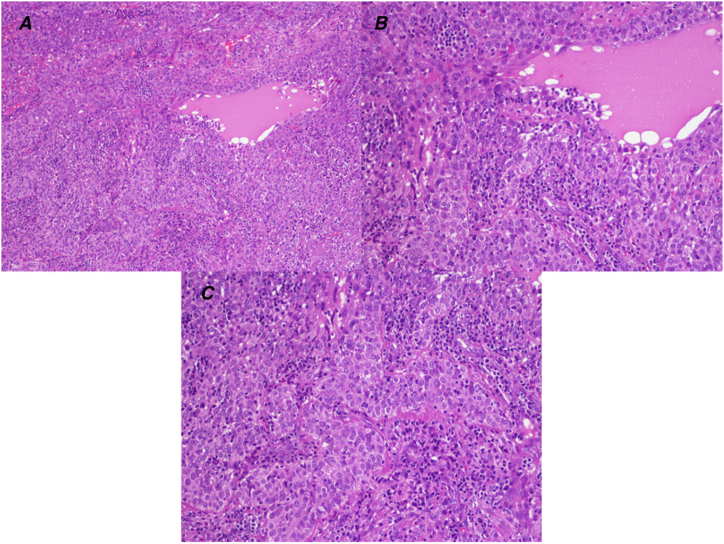
Hematoxylin and eosin staining. A) 10×; B) 20×; C) 20×.

Under local anesthesia, a needle aspiration biopsy of the lesion was performed, from which mainly sero-ematic material was obtained and submitted for thin-prep technique (Fig. 3B), followed by intralesional sampling of gelatinous and loose tissue (Fig. 3C). During the weekly follow-up visits, the patient presented with incomplete healing, characterized by herniation of additional loose parenchymal material. Furthermore, as the swelling continued to grow, it was removed via a diode laser and again sent for pathology. The histological examination revealed a solid astructure neoplasm consisting of epithelial elements with moderate-grade atypia and the presence of mitoses with also some images of atypical mitoses (Fig. 5A, B and C). The immunohistological examination showed neoplastic cells positivity for AE1/AE3, p63, p40, and CK5/6, focally positive for CK7 and negative for CK14, SMA, S-100, CD34, CD99, STAT6, Bcl2, Ber-EP4, and SOX-10. Thus, both samples revealed a secondary location of the B3-type thymoma.

### 2.4 Therapeutic intervention

**Fig. 6:**
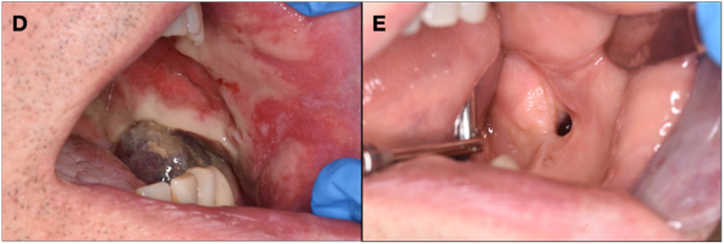
(D) Intraoral view of oral mucositis after radiotherapy; (E) The persisting mucosal fistula after chemotherapy, showing the reduction of the metastasis.

After biopsy, and subsequent check of hemostasis and wound healing after 1 week, the patient was referred to the Oncology Department, where a treatment plan based on Radiotherapy was scheduled. Despite the rapid onset of radiotherapy, it did not appear to have a significant impact on the lesion, causing an important oral mucositis (Fig. 6D). In contrast, chemotherapy with gemcitabine and capecitabine for six cycles achieved excellent results with total regression of the metastatic mass.

However, the healing of the site was incomplete, with persistence of an intra-oral mucosal fistula at the level of the genial mucosa (Fig. 6E).

### 2.5 Follow-up and outcomes

In the subsequent follow-up checks, the patient underwent clinical and radiological examinations. The clinical one confirmed the persistence of a mucosal fistula and a slight fibromucosal thickening at the site of the lesion. Furthermore, approximately 12 months later, the patient underwent a PET-TC for re-staging of the tumor, with the subsequent radiological report regarding the oral cavity. The imaging results show a persistent, heterogeneous increase in metabolic tracer uptake in the left hemimandible, which is associated with changes in the bone structure. This finding suggests ongoing activity in the area that could be linked to various conditions, such as inflammation, infection, or possibly a tumor.

However, the patient reports the disappearance of any algic symptoms.

Clinically, persistence of the mucosal fistula is appreciated. Radiologically, as evidenced by PET-TC, the metabolic tracer uptake is still detected. However, the nuclear physician concluded that this abnormal uptake had a likely inflammatory significance.

In the absence of any existing guidelines, a quarterly clinical-radiological follow-up will be performed, in agreement with the treating oncologist.

At 24-months follow-up, the patient showed no clinical and radiological intraoral signs of local recurrence with reduced tracer uptake in the mandible. Nevertheless, the latest PET-TC revealed a secondary localization of thymoma in the lung. Thus, the patient will undergo a further chemotherapy treatment.

### 2.6 Literature review of similar cases

This review was conducted with a systematic search of the literature in conformity to the Preferred Reporting Items for Systematic Reviews and Meta-Analyses for Scoping Review (PRISMA-ScR) guidelines.

Electronic search was conducted through PubMed, Scopus, and the Cochrane Central Register of Controlled Trials (CENTRAL) database without time filter. The following search strategy was adopted: ((Thymoma) OR (Thymic carcinoma) OR (Malignant Thymoma) OR (Thymus Neoplasms)) AND ((Oral Metastasis) OR (Mandible) OR (Maxilla) OR (Oral Cavity) OR (Distant Metastasis)). Moreover, full-lengths journal articles, reviews, letters, meeting abstracts were also screened through a supplementary manual search, in order to achieve a more comprehensive sight. The last electronic and manual search was conducted on April 13, 2023.

Inclusion criteria were: full-lengths papers; English literature; observational clinical studies (i.e., case reports, perspective and retrospective (both cohort and case-control studies) and randomized clinical trials); patients affected by intraoral metastasis due to thymic tumors. No exclusion criteria were applied.

Study eligibility was conducted by two reviewers (F.S. and C.R.) in an independent and standardized manner, trough an *ad-hoc* extraction forms.

**Fig. 7:**
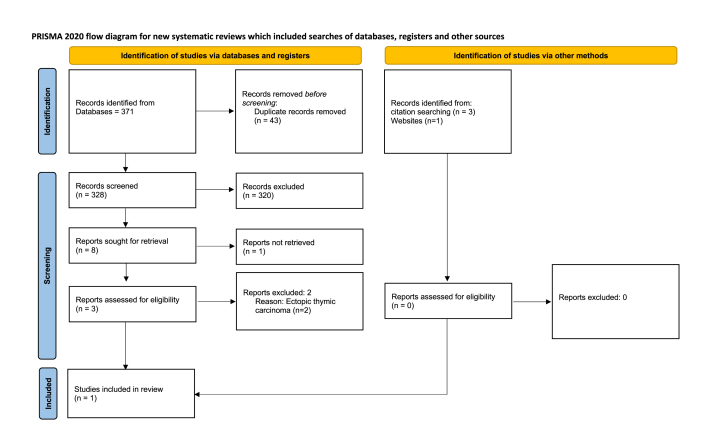
PRISMA for scoping review flow diagram.

The search strategy yielded 328 articles. After the selection process, only 1 article reported an oral metastasis of thymic tumors (Fig. 7) [10]. The reasons of the excluded study were reported in Table 2. The summary of the included study is reported in Table 3.

**Table 2:** Excluded reports.

**Records excluded (nr.)**	**Reason for exclusion**	**Total (nr.)**
325	Off topic. Records did not include thymoma's oral cavity metastasis.	325
2	Reports about ectopic thymic carcinoma of the parotid gland.	327

**Table 3:** Summary of Included reports.

Author	Location	Sex	Age	Intraoral site of metastases	Clinical features	Histological features - Immunohistochemistry	Radiological examination	Timelapse between primary tumor and metastases	Treatment	Course
Ichino et al., 1983 [10]	Kumamoto University Hospital, Department of Otolaryngology	Female	46 y.o.	Right Mandible	Encapsulated tumor, partially adhesive and elastic soft mass. Size: 6 × 5 × 4 cm	Primary tumor: Lymphoid type metastatic thymoma of the mandibula.	Bone scintigraphy with ^99m^Tc-diphosphonate showed increased uptake in the skull, left mandibula, 12th dorsal vertebra and left femur	1 year	Surgery, Radiation therapy. Chemotherapy	Died after 4 years, due to chemotherapy complications and lung infection
						Metastasis: Epithelial type thymoma				
Scilla et al., 2024	Fondazione Policlinico Universitario Agostino Gemelli, Rome, Italy	Male	62 yo	Left Mandible	Non-ulcerated, hard-elastic and completely enclosed within the epithelium swelling. No pain. Bluish in color. Size: 3cm × 2cm × 2 cm	B3-type thymoma.	CT dentascan revealed a large osteolytic lesion.	2 years	Radiation therapy. Chemotherapy	2 years follow-up with no oral local recurrence. Patient developed lung metastasis.
						Positivity for AE1/AE3, p63, p40, and CK5/6, focally positive for CK7 and negative for CK14, SMA, S-100, CD34, CD99, STAT6, Bcl2, Ber-EP4, and SOX-10.				

## 3 Discussion

Thymic epithelial tumors (TETs) are composed of thymic epithelial tumor cells and normal lymphocytes. The average age at diagnosis is 50–60 years, and no risk factors have been identified. The etiopathogenesis of thymic carcinoma and thymoma can be traced to abnormal proliferation of a group of epithelial lining cells in the thymus, known as reticuloepithelial cells and generally exhibits slow growth [14,15]. With regards to their epidemiology, thymic tumors are considered rare, representing less than 1 % of tumors that affect humans [20]. Thymoma is frequently associated with other diseases related to the immune system, such as myasthenia gravis, multiple autoimmune syndromes, pure red cell aplasia, systemic lupus erythematosus, and some forms of autoimmune diseases of the oral cavity, such as oral lichen planus, which often presents in the erosive variant [14; [[16], [17], [18], [19]].

This paper provides a rare case of oral metastasis of thymoma with a rapidly worsening trend in a 62-year-old Italian male patient with no other comorbidities. Clinically, the swelling was non-ulcerated and completely enclosed within the epithelium located in the left mandible, at the level of the mandibular angle in the retromolar trigone area, was, in the absence of pain, neither spontaneous nor provoked, and had a hard-elastic consistency and bluish in color (Fig. 3). Although the patient reports lip numbness of the left lower and upper hemilabra, approximately 55 % of patients are asymptomatic at the time of diagnosis, and thymoma is usually an incidental finding. In contrast, only about 15 % of symptoms related to tumor paraneoplastic syndromes, such as myasthenia gravis, prevail [21]. Symptomatic thymomas usually involve local compression. In other cases, dyspnea, cough and chest pain may be the presenting symptoms [22]. In the presence of an anterior mediastinal lesion, the diagnosis of thymoma is usually clinically suspected based on the presence of myasthenia gravis or other associated syndromes, such as autoimmune disease, endocrine abnormalities and inappropriate antidiuretic hormone syndrome (SIADH) [23].

Metastases are usually confined to the pleura, pericardium, or diaphragm, whereas extrathoracic metastases are uncommon although we reported a rare case of intraoral metastasis [24]. Nearly 30 % of thymomas are inoperable, resulting in 5-year survival rates of 36–53 % [25]. Inoperable, metastatic thymomas are typically managed with chemotherapy to control tumor-related symptoms; no prolonged survival is usually expected [26]. Although our patient underwent a thymectomy, after 2 years sopravlavicolar metastatic lymphnodes and intraoral metastasis were detected, and after 4 years a lung metastasis was detected, thus the patient underwent a further chemotherapy treatment.

### 3.1 Discussion of the relevant medical literature

To the best of the authors' knowledge, the present case report is the second one to report an oral metastasis of thymoma confirmed by a histological assessment. The first report of oral metastasis of thymoma was described by Ichino et al. (1983) in a 46yo woman [10]. The patient suffered by a metastatic thymoma of the mandible that occurred after 1 years from primary oncological diagnosis. The patient was followed up 48 months until death, which occurred due to chemotherapy's adverse reaction and lung infection. The summary of the included study in this scoping review is reported in Table 3.

Through the literature review, the search identified also two reports of ectopic thymic carcinoma of the parotid gland were found that were excluded since thymic carcinoma represents a different neoplasm originating from thymic cells [12,13].

It deserves to be reported that during the abstract selection, another study conducted by Piller P et al. (1993) reported a thymoma metastasis, nevertheless the full text of the article was in French and unavailable online and in digital archives [11]. Moreover, the authors of the article were contacted via ResearchGate, but they never provided a response. Thus, the study was excluded.

### 3.2 Strengths and limitations in approach to this case

The rapid diagnosis of the lesion, which occurred within a few days of the patient's initial visit, and the decision to immediately perform a biopsy of the lesion and represent the main strengths of the authors' approach to the case, allowing a proper therapy.

However, oral metastases due to thymoma are an extremely rare event, and consequently there is an obvious absence of therapeutic guidelines, which led to the choice of a treatment based on radiotherapy, and subsequently on chemotherapy, borrowed from guidelines for the treatment of distant thymic metastases.

After conducting in-depth research and analysis, it is apparent that patients with thymoma should be visited by an oral medicine specialist, in order to early diagnose any oral manifestation of thymoma. In fact, research suggests that thymoma may also be linked to the development of autoimmune diseases, including oral lichen planus [27].

Additionally, visiting patients with thymoma to assess oral lichen planus can also provide additional valuable insights into the relationship between thymoma and autoimmune diseases.

### 3.3 Differential diagnoses

In the oral cavity, the appearance of a non-ulcerated lesion with rapid growth warrants differential diagnosis with several benign and malignant diseases.

An initial differential diagnosis was made with a dental abscess that had infected a pre-existing cyst. Similarly, osteomyelitis, a bone infection that can occur due to trauma, dental infections, or hematogenous spread, was considered in the differential diagnosis. It can cause bone destruction, pus formation, and sequestration in the maxillary bones [28]. However, these diagnoses were discarded due to the absence of typical signs and symptoms: the patient reported no pain, there were no neighboring teeth and there were no signs of infection (i.e., absence of discharge of purulent material, absence of fever) [29].

The appearance of the lesion could also suggest a large mucocele or sialolithiasis of the major glands; However, this option was immediately ruled out after consulting the radiographic examination, as these two conditions do not present with mandibular localization [30].

Maxillary cysts and odontogenic tumors were included in the differential diagnosis, especially because of the presence of an osteolytic lesion. For example, odontogenic keratocysts and locally invasive ameloblastomas, which are frequently localized in the mandible, were considered in the differential diagnosis. Rare cases of keratocysts have been described peripherally in the context of gingival soft tissues [31,32]. Most ameloblastomas are located in the maxillary bones, however in some cases they can be found in the gingival soft tissues. In the latter case, they are called peripheral or extraosseous ameloblastomas [33]. The differential diagnosis was obtained through histological diagnosis, and, in addition, the rapid growth in the soft tissues of the patient's lesion was not typical of odontogenic cysts and tumors.

Some other lesions of the maxillary bones were also considered in the differential diagnosis of this non-ulcerative swelling. Osteoma, is a benign tumor that can present in any area of the body, including the maxillary bones. It generally appears as a painless mass in the bone that can cause facial asymmetry, displacement of dental elements, and limited mouth opening [34]. We ruled it out because the patient's lesion in question grew too rapidly and because the radiographic margins of osteoma are usually sharp with a surrounding area of osteosclerosis [34].

Nevertheless, the clinic-radiographic features of the lesion, especially its rapid growth, immediately led the authors to suspect a malignancy: in particular, lymphoma was considered: lymphomas are malignant neoplastic proliferations of the immune cells. Moreover, the most common site of extra-nodal lymphomas is the gastrointestinal tract, followed by head and neck district [35]. Differential diagnosis with lymphoma was possible only because of the histological examination. Moreover, both benign and malignant connective tissue lesions (i.e., Langerhans cell histiocytosis, juvenile xanthogranuloma, sarcoma) should be considered in the differential diagnosis. Thus, biopsy is mandatory to achieve a correct diagnosis, and immune-histochemistry analysis can lead to a final diagnosis [36].

However, the patient's medical history soon led to a diagnostic doubt regarding oral cavity metastasis, which was subsequently confirmed by histological examination.

## 4 Conclusions

This paper provides a rare case of oral metastasis of thymoma that represent an almost undescribed condition, as highlighted by the review of published cases. It is important to emphasize the importance of oral screening of patients with a diagnosis of neoplastic pathologies of the thymus, considering the relationship between these diseases and oral disorders. Future reports will be useful in increasing knowledge regarding this area.

## CRediT authorship contribution statement

**Francesco Scilla:** Writing – original draft, Visualization, Methodology, Data curation. **Cosimo Rupe:** Writing – original draft, Validation. **Gioele Gioco:** Validation, Supervision, Formal analysis. **Luca Raffaelli:** Visualization, Validation. **Filippo Lococo:** Writing – review & editing, Visualization. **Ciro Mazzarella:** Writing – review & editing, Visualization. **Guido Rindi:** Writing – review & editing, Visualization. **Romeo Patini:** Writing – original draft, Validation. **Carlo Lajolo:** Writing – original draft, Visualization, Validation, Conceptualization.

## Patient consent statement

The patient signed an informed consent form to allow the publication of this paper.

## Data availability

The data that support the findings of this study are available from the corresponding author upon reasonable request.

## Funding

This research did not receive any specific grant from funding agencies in the public, commercial, or not-for-profit sectors.

## Declaration of competing interest

The authors declare that they have no known competing financial interests or personal relationships that could have appeared to influence the work reported in this paper.
